# OPTIMAS-DW: A comprehensive transcriptomics, metabolomics, ionomics, proteomics and phenomics data resource for maize

**DOI:** 10.1186/1471-2229-12-245

**Published:** 2012-12-29

**Authors:** Christian Colmsee, Martin Mascher, Tobias Czauderna, Anja Hartmann, Urte Schlüter, Nina Zellerhoff, Jessica Schmitz, Andrea Bräutigam, Thea R Pick, Philipp Alter, Manfred Gahrtz, Sandra Witt, Alisdair R Fernie, Frederik Börnke, Holger Fahnenstich, Marcel Bucher, Thomas Dresselhaus, Andreas PM Weber, Falk Schreiber, Uwe Scholz, Uwe Sonnewald

**Affiliations:** 1 Leibniz Institute of Plant Genetics and Crop Plant Research (IPK), 06466 Stadt Seeland, Corrensstr. 3; 2 Department of Biology, Friedrich-Alexander University of Erlangen-Nuremberg, 91054 Erlangen, Staudtstr. 5, Germany; 3 University of Cologne, Botanical Institute, 50923 Köln, Albertus-Magnus-Platz, Germany; 4 Plant Biochemistry, Heinrich-Heine-University, Universitätsstr. 1, 40225 Düsseldorf, Germany; 5 International Graduate Program for Plant Science (iGrad-plant), Heinrich Heine University Düsseldorf, 40225 Düsseldorf, Germany; 6 Cell Biology and Plant Biochemistry, University of Regensburg, Universitätsstr. 31, 93040 Regensburg, Germany; 7 Department of Molecular Physiology, Max Planck Institute of Molecular Plant Physiology, 14476 Potsdam-Golm, Am Mühlenberg 1, Germany; 8 metanomics GmbH, 10589 Berlin, Tegeler Weg 33, Germany; 9 Martin Luther University Halle-Wittenberg, Institute of Computer Science, 06120 Halle, Von-Seckendorff-Platz 1, Germany

**Keywords:** Maize, *Zea mays*, Database, WGCNA, Biomass, Yield, Data integration, Transcriptomics, Metabolomics, Phenomics

## Abstract

**Background:**

Maize is a major crop plant, grown for human and animal nutrition, as well as a renewable resource for bioenergy. When looking at the problems of limited fossil fuels, the growth of the world’s population or the world’s climate change, it is important to find ways to increase the yield and biomass of maize and to study how it reacts to specific abiotic and biotic stress situations. Within the OPTIMAS systems biology project maize plants were grown under a large set of controlled stress conditions, phenotypically characterised and plant material was harvested to analyse the effect of specific environmental conditions or developmental stages. Transcriptomic, metabolomic, ionomic and proteomic parameters were measured from the same plant material allowing the comparison of results across different omics domains. A data warehouse was developed to store experimental data as well as analysis results of the performed experiments.

**Description:**

The OPTIMAS Data Warehouse (OPTIMAS-DW) is a comprehensive data collection for maize and integrates data from different data domains such as transcriptomics, metabolomics, ionomics, proteomics and phenomics. Within the OPTIMAS project, a 44K oligo chip was designed and annotated to describe the functions of the selected unigenes. Several treatment- and plant growth stage experiments were performed and measured data were filled into data templates and imported into the data warehouse by a Java based import tool. A web interface allows users to browse through all stored experiment data in OPTIMAS-DW including all data domains. Furthermore, the user can filter the data to extract information of particular interest. All data can be exported into different file formats for further data analysis and visualisation. The data analysis integrates data from different data domains and enables the user to find answers to different systems biology questions. Finally, maize specific pathway information is provided.

**Conclusions:**

With OPTIMAS-DW a data warehouse for maize was established, which is able to handle different data domains, comprises several analysis results that will support researchers within their work and supports systems biological research in particular. The system is available at http://www.optimas-bioenergy.org/optimas_dw.

## Background

Maize is a major crop plant, grown for human and animal nutrition, as well as a renewable resource for bioenergy. Considering that fossil fuels are limited, it is clear that there must be alternative ways of production. Biofuel might be such an alternative. When looking at the large increase of the world’s population it is also obvious that more people need to be provided with food, but in contrast there is less arable land available. The world’s climate change causes more extreme weather conditions all over the world, which means that plants must be more resistant to such conditions. Therefore, it is important to find ways to increase the yield and biomass in maize plants and to study how maize plants react within specific abiotic and biotic stress situations. The OPTIMAS project (OPTImisation of bioMASs in maize) was started in 2009 to find answers to the question of yield and biomass increase and furthermore to obtain useful insights into the distribution of plant resources between vegetative biomass and corn yield ( http://www.optimas-bioenergy.org/).

The rapid improvement of analytical methods now enables to extend systems biology approaches directly for crop plant systems. During the project maize plants were grown under a large set of controlled stress conditions, characterised phenotypically, and plant material was harvested to analyse the effect of specific environmental conditions (e.g. cold, drought or nutrient stress) or developmental (e.g. flowering, leaf gradient dependent growth stage) stages. It was anticipated that the collected measurement data of transcriptomics, metabolomics, ionomics, and proteomics from the same plant material would facilitate the comparison of results on different omics domains. A better understanding of metabolic events underlying phenotypic changes will allow to further optimise maize breeding and cultivation for its multiple purposes as food, feed and bioenergy source. A central goal was to store the collected data in a database and to find a concept to link these data from the different domains and finally to provide access to all collected data and analysis results to the users.

There are already several maize databases available such as MaizeGDB [1] and Panzea [2]. MaizeGDB is a database for storing and curating genetics and genomics related data of maize. It serves also as a community platform comprising maize references and information about persons and organisations. Panzea is a database dealing with molecular and functional diversity in the maize genome. The database includes genotypic, phenotypic and polymorphism data.

Here OPTIMAS-DW, a comprehensive data warehouse containing large amounts of integrated maize-specific data from five domains is presented. A data warehouse is a database which enables a user to integrate and analyse data from different data domains. This includes transcriptomic, metabolomic, proteomic, ionomic and phenomic data as well as metabolic pathways. So far there exists no other database allowing to store data of all these data domains. OPTIMAS-DW is a public information resource which provides researchers with a large collection of data for their own research. This paper describes the structure and usage of OPTIMAS-DW.

## Construction and content

### Database introduction

**Figure 1:**
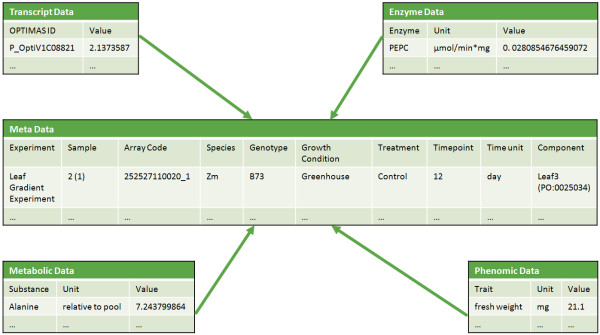
**OPTIMAS Metadata Concept Example.** Data from different data domains are linked through metadata. The concept enables a user to get data from different data domains with specific characteristics of an experiment. In this example the metadata contains a sample of the nitrogen stress experiment. The measurement values are linked to these metadata. With this approach the user can for example extract the information, that in leaf 3 of sample 2(1) a fresh weight of 21.1 mg was measured.

OPTIMAS-DW is accessible via a web application. This allows the user to get access to all data collected within the OPTIMAS project. It is based on ORACLE Application Express (APEX) technology. The web interface is a two level tab system including more than 60 pages to present the content of OPTIMAS-DW. The data itself are stored in an ORACLE database. The database schema comprises around 60 tables to store the raw data as well as meta information and analysis results. The challenge of storing the data is the existence of different data domains which have to be linked. Here we could used experiences made in previous projects [3]. In our method, the database schema includes metadata describing the experiments. Each measurement value can be connected to a specific sample which has specific characteristics such as genotype, plant growth stage, treatment or plant anatomy. An example of the concept is given in Figure 1. In Additional file 1 an excerpt of the OPTIMAS database schema is shown. It illustrates how the concept is realised inside the database. The key table is *t103_measurement_value*, where the data domain specific schema is connected to the metadata schema. All data domain schemas currently available in OPTIMAS-DW are included into the file to illustrate that the system could be enhanced by different kind of data domains.

**Figure 2:**
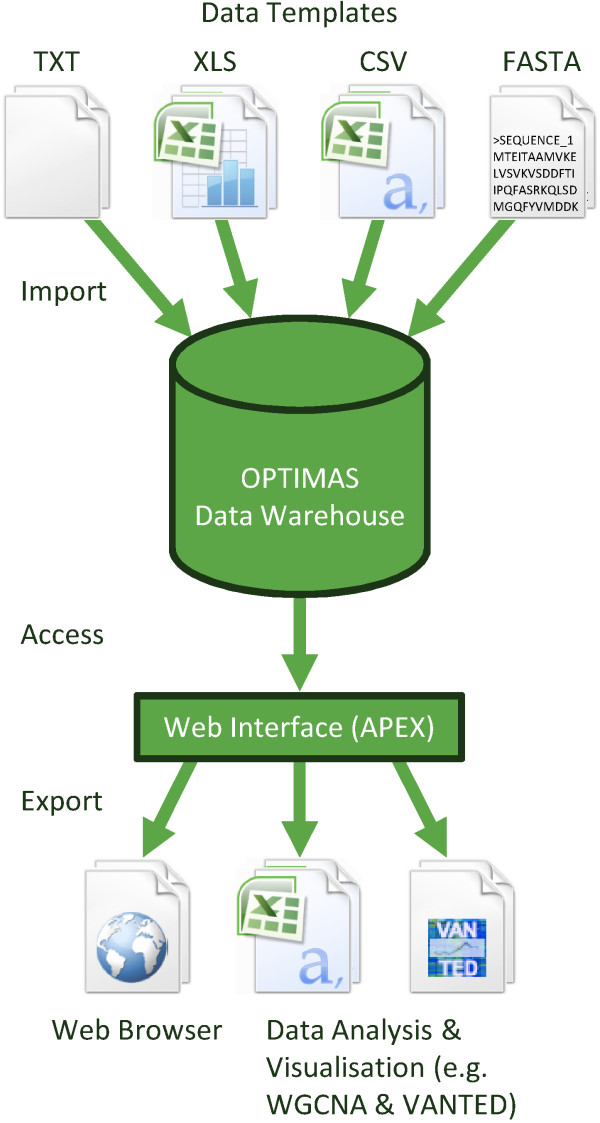
**OPTIMAS Data Pipeline.** Experimental data are collected with different templates which are imported by a Java based import tool into the OPTIMAS Data Warehouse. Using a web interface the data can be exported for further data analysis (e.g. with WGCNA [6]) and visualisation (e.g. with VANTED [4]).

The general pipeline of OPTIMAS-DW for import, storage and retrieval of OPTIMAS data is illustrated in Figure 2. Depending on the data domain the information is provided in different formats. Sequence data is provided in fasta format. Transcriptomic data is available in formated text files. Metabolomic, ionomic, proteomic and phenomic data are provided using an Microsoft Excel based template. Additionally, metadata are provided within this template including the specific characteristics mentioned before to describe each biological entity within an experiment. Furthermore, information about the array code and a sample name is provided to connect the data to the related transcript data. The data are imported into the database by an import tool developed with the Java programming language. During the import procedure the vocabulary used, is checked as well as the correctness of datatypes to ensure that only valid data are imported into the OPTIMAS data warehouse.

The web application itself enables the user to browse, search, filter and download stored data. OPTIMAS-DW supports two ways of data export: (a) the export to a tab delimited file comprising all data selected by the user (e.g. data filtered for a specific treatment or a timepoint) and (b) the export into a special format for data analysis and visualisation in VANTED [4], a tool for the **V**isualisation and **A**nalysis of **N**e**t**works containing **E**xperimental **D**ata. The export tool is a Java Web Start application where the user selects the file format and a target directory where the file will be stored. Using VANTED the user is able to map the experimental data from the OPTIMAS data warehouse onto maize specific pathways stored in MetaCrop [5], a manually curated repository comprising high quality data about crop plant metabolism. The pathways can also be accessed through the web interface of OPTIMAS-DW. Therefore the MetaCrop database schema provides the relevant information to link the web interface of OPTIMAS-DW directly to the specific pathways in MetaCrop. Besides using VANTED, the data can also be analysed with other tools, such as an R package called WGCNA (Weighted Gene Correlation Network Analysis) [6]. WGCNA is a widely used tool [7-9], allowing the user to find interesting genes responsible for high biomass production in maize plants. The results of these analyses can be accessed through the web interface. Beside the WGCNA analysis results, GeneSpring (version GX11) analysis results are available as well.

### Array design

Within the OPTIMAS project a 44K Agilent oligo chip was designed to perform different gene expression analyses. Based on experiences from previous works with the POCI array (Potato Oligo Chip Initiative) [10], the database schema from this project was adapted for the OPTIMAS oligo chip. Therefore at the start of the project the newest available maize genome version 3b.50 ( http://maizesequence.org) was used to select the unigenes for the oligo chip. BLAST [11] analyses provided information about redundancies within the dataset. To overcome the redundancies a MIRA [12] assembly (Mimicking Intelligent Read Assembly) was performed. Furthermore, 17,723 contigs and 16,881 singletons were selected as unigenes, and four additional sequences, special candidate genes from project partners, which were not present in the selected unigenes, were added. To achieve a number of 42,000 unigenes, 7,392 EST sequences from NCBI maize unigenes, which had no hit on the predicted maize genes, were selected additionally. The eArray-software from Agilent ( http://www.genomics.agilent.com) computed a number of 41,780 60-mer oligos from the unigenes, which can be browsed and downloaded at OPTIMAS-DW. When the maize genome was published in November 2009 [13], the quality of the 44K chip was analysed in comparison to the maize genome version 4a.53. It was discovered, that 52,040 of the 53,764 gene models got a hit in the OPTIMAS unigene set. Therefore, it was decided to generate a mapping from the OPTIMAS identifiers to the 4a.53 version of the maize genome. Beside the availabilty in OPTIMAS-DW the chip data is published in GEO [14] and is available online ( http://www.ncbi.nlm.nih.gov/geo/query/acc.cgi?acc=GPL14913). As an additional service, OPTIMAS-DW provides ViroBLAST [15], which enables users to run a BLAST of sequences against the OPTIMAS unigene set, the maize genome (version 3b.50 and 4a.53) or the NCBI maize unigene set.

### Annotation

Several BLAST results are available in OPTIMAS-DW. For example, a Blast2Go [16] is supported to connect sequence data with Gene Ontology annotations [17] and Enzyme Commission numbers (EC) [18]. Therefore a BLAST of the OPTIMAS unigene set against the maize genome version 4a.53 and a further BLAST from the maize genome version 4a.53 to NRPEP was carried out and then mapped to the Gene Ontology terms. A complete list of available BLAST results is shown in Table 1. Furthermore, a mapping file to link the OPTIMAS identifiers to the gene identifiers from the maize genome version 4a.53 is provided. Additionally, a mapping vice versa from the maize genome version 4a.53 to the best hit OPTIMAS identifier enhanced by annotations from TAIR10 [19] as well as gene ontology and KOG description based on Sorghum data [20] (sbi1.4 annotation) is provided.

**Table 1:** Overview about BLAST analyses in OPTIMAS-DW

**BLAST type**	**Query database**	**Hit database**	**Entries in hit database**
BLASTX	Maize Genome 3b.50	NCBI non redundant Peptides	7,987,196 proteins
BLASTN	Maize Genome 3b.50	NCBI Zea mays Unigene Build 75	82,630 ESTs
BLASTN	Maize Genome 3b.50	EMBL fungi ESTs	2,028,363 ESTs
BLASTX	Maize Genome 4a.53	NCBI non redundant Peptides	7,987,196 proteins
		(used for Blast2Go)	
BLASTX	OPTIMAS Oligo Set	Uniref, version 2011-09-21	

### Data overview

Within the OPTIMAS project several treatment and plant growth stage experiments were performed and information from different data domains was collected. Within the treatment experiments maize plants were grown under specific stress conditions, such as nitrogen stress, cold stress, and drought stress to discover how the plants behave under those conditions. Studies on mycorrhizal maize lines were performed as well. The plant growth stage experiments include leaf gradient and flowering time analysis of maize plants. The collected data comprise transcriptomic, metabolomic, ionomic, proteomic and phenomic data. An overview about the stored experiments is given in Table 2.

**Table 2:** Overview about experiments and measurements for all data domains stored in OPTIMAS-DW

**Experiment**	**Transcript**	**Metabolic**	**Ionomic**	**Enzyme**	**Phenomic**
	**data**	**data**	**data**	**data**	**data**
**Treatment experiments**					
Cold Stress (A188 and B73)	4,010,880	28,769	1,140	-	208
Drought Stress	2,005,440	39,082	-	-	44
(study of 2 pairs of maize inbred lines,					
each having one line with a good					
water-use-efficiency and one line					
with a poor one)					
Nitrogen Stress (A188 and B73)	2,673,920	14,629	999	-	176
Nitrogen Use Efficiency 1	-	61,035	-	-	1,699
(16 maize inbred lines)					
Nitrogen Use Efficiency 4	-	-	-	-	2,312
(C:N ratios of different plant parts)					
Mycorrhiza Compartment 2/3	501,360	7,510	438	-	108
(Physiological, elemental, gene					
expression and metabolite analysis					
of mycorrhizal maize line B73)					
Mycorrhiza Compartment 6/8	-	-	4,654	-	1271
(Screening of 27 maize lines for their					
responsiveness towards the arbuscular					
mycorrhiza fungi by physiological and					
elemental analysis)					
Mycorrhiza Compartment 9	-	-	5,699	-	407
(Analysis of 2 closely related pairs					
of maize lines for their physiological,					
elemental and metabolite profile in					
reaction to mycorrhiza infection.)					
Field Experiment 2010	-	169,991	-	-	3,073
(26 inbred lines grown in the field)					
^13^C Disc feeding (^13^C enrichment)	-	1,152	-	-	-
^13^C Glucose feeding (^13^C enrichment)	-	286	-	-	-
^13^CO2 feeding (^13^CO2 enrichment)	-	743	-	-	-
^15^N Urea feeding (^15^N enrichment)	-	351	-	-	-
**Plant growth stage experiments**					
B73 Grains	-	-	207	-	-
(Comparison of elemental composition					
of maize kernels of line B73 provided					
by Regensburg or BASF)					
Flowering Time	1,504,080	27,823	-	-	-
(analysis of 2 pairs of maize inbred					
lines to identify transcripts/metabolites					
regulating flowering time in maize)					
Leaf Gradient	1,671,200	16,491	720	180	30
(analysis of the developmental					
gradient of the third maize leaf)					
Complete Data Warehouse	12,366,880	367,862	13,857	180	9,328

## Utility

**Figure 3:**
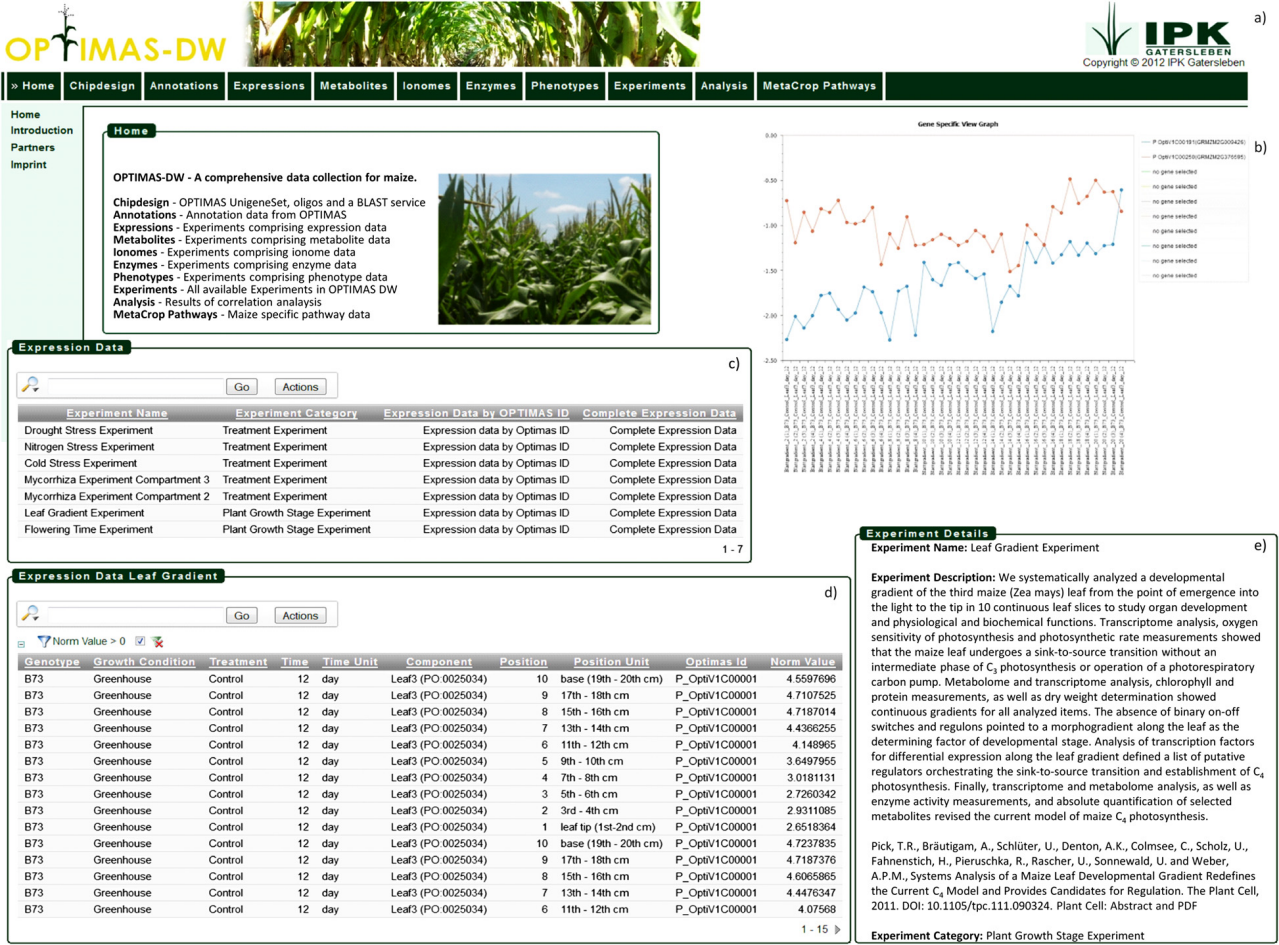
**OPTIMAS-DW Compilation of Screenshots.** A compilation of screenshots from OPTIMAS-DW. **a**) Navigation comprising all data domains and functions. **b**) Gene specific view graph for gene expression visualisation. **c**) Overview of experiments containing transcript data. **d**) Browsing and filtering experimental data. **e**) Descriptions for each experiment are available.

In all experiments, data from different domains were collected and can be accessed separately through the web interface of OPTIMAS-DW (Figure 3a). When choosing for example the transcriptomic data domain the user can browse all experiments containing transcriptomic data (Figure 3c). The user can now consider to see gene specific data or to navigate through the whole transcriptomic dataset of an experiment. Within the other data domains the user can proceed the same way. Metabolomic data can be filtered by a specific substance name, ionomic data can be accessed by filtering for one specific ion name, proteomic data can be filtered by a specific enzyme name and phenomic data can be filtered by a specific trait. The transcriptomic data domain additionally comprises a gene specific view enabling the user to inspect and compare gene expressions of up to ten selected genes (Figure 3b). The view allows the user to filter by experiments and to determine the order of the experiment samples.

Another way to navigate through the data starts at the experiment view. Here, the user can browse all experiments and can access the data from all domains available for a particular experiment sample. As described in Figure 1, the metadata concept is realised within this web page. By the selection of a specific experiment sample, data from each data domain can be retreived. When using the data domain specific view instead, the metadata is also visible to the user but only domain specific data can be browsed. The domain specific view therefore enables the user to retrieve and analyse domain specific data while the experiment view has the advantage to retrieve and analyse data of different domains of specific samples. For each experiment also a short description is availble (Figure 3e).

**Figure 4:**
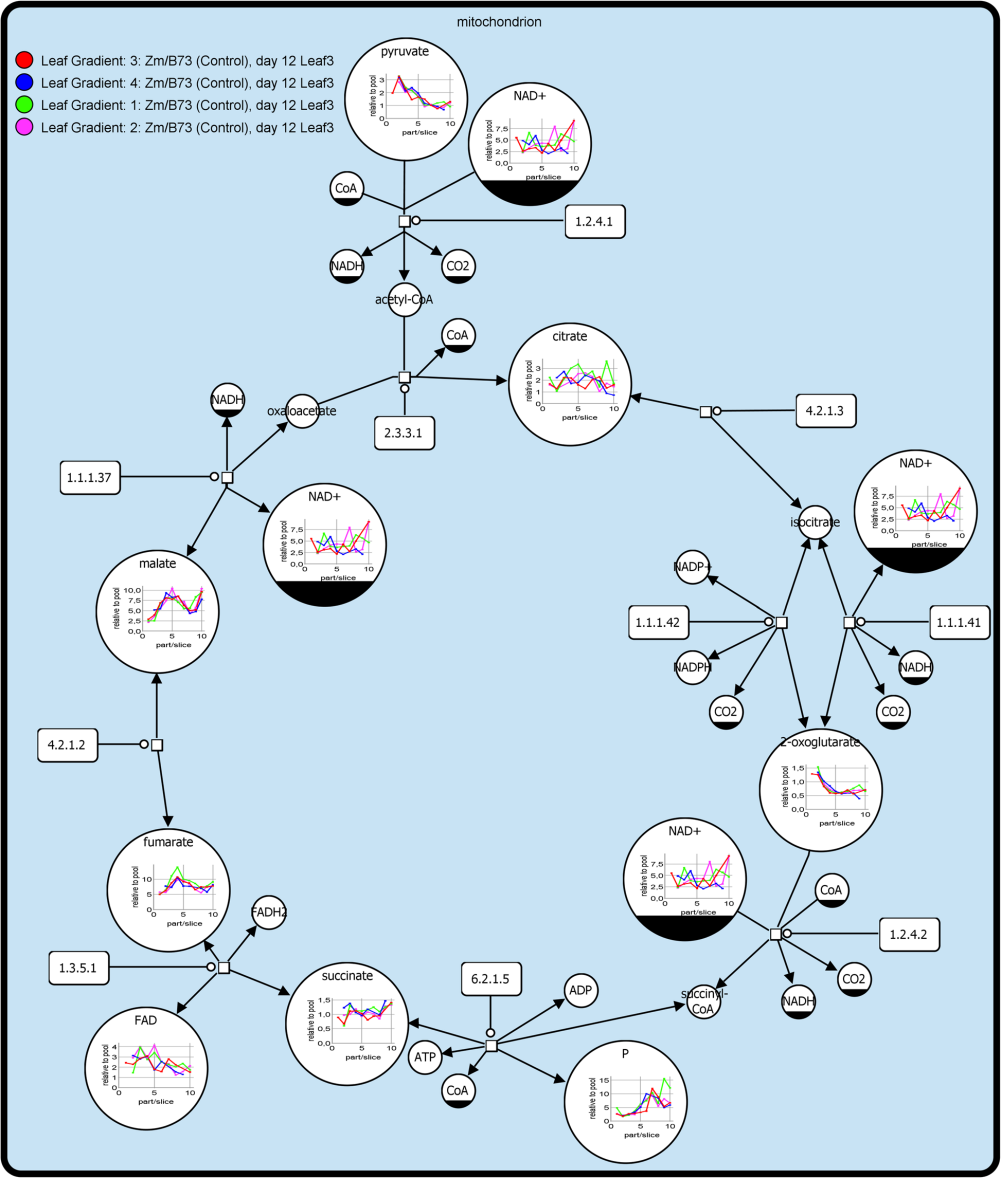
**VANTED Data Visualisation Example.** Measured data from experiments stored in OPTIMAS-DW can be mapped onto pathways stored in MetaCrop [5] by using the VANTED [4] software. It enables the user to visualise the data and to perform further data analysis. The map is visualised by the Systems Biology Graphical Notation (SBGN) [23]. The small squares represent chemical reactions. The reactions are catalysed by enzymes represented as rectangular containers with rounded corners. The catalysis is represented by a small empty circle. The metabolites are illustrated as circular containers and are either reactant or product of a reaction. When a metabolite occurs multiple times it is decorated with a clone marker (e.g. NAD^+^).

One of the experiments stored in the OPTIMAS-DW is the leaf gradient experiment. Within the leaf gradient experiment a systematic analysis of a developmental gradient of the third maize leaf was accomplished to study organ development as well as physiological and biochemical functions [21]. Data from different data domains were measured and stored in the data warehouse. In detail there are transcriptomic, metabolomic, ionomic, proteomic and phenomic data available. The number of measurement values for this experiment is given in Table 2. As mentioned in the background section, data were measured from the same plant material, enabling us to directly correlate the data from different domains. In the leaf gradient experiment for example correlations between transcripts and enzyme activity as well as correlations between metabolites and enzyme activity could be detected. In Figure 3d a report comprising a selection of transcriptomic data for the leaf gradient experiment is shown. Here the data was filtered for upregulated genes. Figure 4 illustrates an example of a data mapping with VANTED (method described in Junker et al. [22]). Metabolomics data from the leaf gradient experiment were mapped onto the TCA-Cycle. For each metabolite that was measured in the experiment the corresponding node in the graph includes a line chart visualising the data. Here, the user can see the measured metabolite concentration in each part of the third leaf from tip (1) to base (10).

Beside the experimental data the user is able to retrieve analysis results from the web interface. This includes WGCNA results where specific conditions were defined. The user can, for example, detect genes that are highly correlated to fresh weight, growth rate or metabolite profiles in the nitrogen stress experiment [24]. Additionally, the chip ID and the correlation values are listed as well as the annotation. Furthermore, module lists of a WGCNA analysis are available where the transcript data of all experiments were included. A module is a cluster of interconnected nodes representing highly correlated genes. Here, the user can detect which genes are located in the same module. Additionally, a gene expression profile for each module is available visualising the average expression pattern (module eigengene) for each sample of analysed experiments. The WGCNA module overview enables the user to carry out in which module a specific gene is located in the different WGCNA analysis results. With the help of that function the user can detect genes, which will react in a different way in different experiments.

Finally, the user can browse through the maize specific pathways stored in MetaCrop. A table provides a list of these pathways including clickable thumbnail images redirecting the user to MetaCrop. In MetaCrop the user can navigate through the pathway data to get detailed information.

## Discussion

OPTIMAS-DW provides an innovative concept to link data from different data domains through metadata. It is very easy to extend the data warehouse by additional data domains by adjusting the main components of OPTIMAS-DW, such as data templates, the import tool, the database schema, and the web interface. With the web interface the user can extract data very easy either by browsing through a specific data domain of interest or by looking for data related to a specific experiment. Tools like the Gene Specific View or the WGCNA analysis enable the user to get answers to different systems biological questions. With WGCNA it is for example possible to detect genes, which are correlated to the growth rate or the fresh weight of a maize plant. In that case transcript and phenomic data is used by the analysis to detect responsible genes for biomass increase in maize plants. With OPTIMAS-DW the user is able to perform data analysis using different data domains. By using the Gene Specific View the user is, for example able to look at the behavior of genes of interest in different experiments and conditions such as genotype, plant growth stage, treatment or plant anatomy.

OPTIMAS-DW enables us to store more experimental data in future maize related projects to enhance our data collection of maize. By further extending the database content and its functionality OPTIMAS-DW could help the researchers to better understand the systems biological processes in maize plants. Because of the experiences gathered during the development of OPTIMAS-DW, the infrastructure and pipeline could also be used to set up data warehouses for other plant organisms. Furthermore, ways of data analysis could be improved in the future enabling users to start data analyses directly from the web interface and with selected data of their interest.

## Conclusions

With OPTIMAS-DW a comprehensive data warehouse for maize was established, which is able to handle different data domains and which comprises several analysis results that will support researchers within further projects. The easy access to transcriptomics, metabolomics, ionomics and proteomics data from plant material with detailed phenotypic description allows the use of the full potential of large scale analysis tools in the future. It is also possible to continuously extend the data warehouse by adding more experimental data, even in data domains which are not already available in OPTIMAS-DW. The concept to combine different data domains by metadata will be used in future projects. We believe that OPTIMAS-DW will be a very valuable public data warehouse for maize related research and supports systems biological research in particular.

## Availability and requirements

The OPTIMAS-DW is available with no restrictions at the following http://www.optimas-bioenergy.org/optimas_dw. All datasets are free to use and can be downloaded via the web interface. There are no restrictions on use of the database as well as all stores data sets.

## Competing interests

The authors declare that they have no competing interests.

## Author’s contributions

CC developed the data warehouse including the OPTIMAS-DW web application. CC, TC and AH designed and provided the data import template. U. Schlüter, NZ, AB, TRP, PA, MG, SW and HF provided the experimental data. MM performed the WGCNA analyses. ARF, FB, HF, MB, TD, APMW, and U. Sonnewald supervised the biological part of the project. FS and U. Scholz supervised the bioinformatics part of the project. U. Sonnewald headed the project consortium. All authors tested and used OPTIMAS-DW and thereby contributed to the improvement of the web interface usability. All authors read and approved the final manuscript.

## Supplementary Material

Additional file 1 **OPTIMAS Database Schema.** The OPTIMAS Database Schema can be divided into two main parts, the metadata on the one side and the data domain schemas on the other side. The metadata and each data domain schema are linked through table optimas.t103_measurement_ value. The primary key of the data domain entry is stored in t103_ measurement_value_id while the information about schema and table is stored in the related tables. Click here for file
