# Supplementary figures and images for: OPTIMAS-DW: A comprehensive transcriptomics, metabolomics, ionomics, proteomics and phenomics data resource for maize
Source: BMC Plant Biol. 2012 Dec 29;12:245. doi: 10.1186/1471-2229-12-245 (PMC3577462; doi:10.1186/1471-2229-12-245)

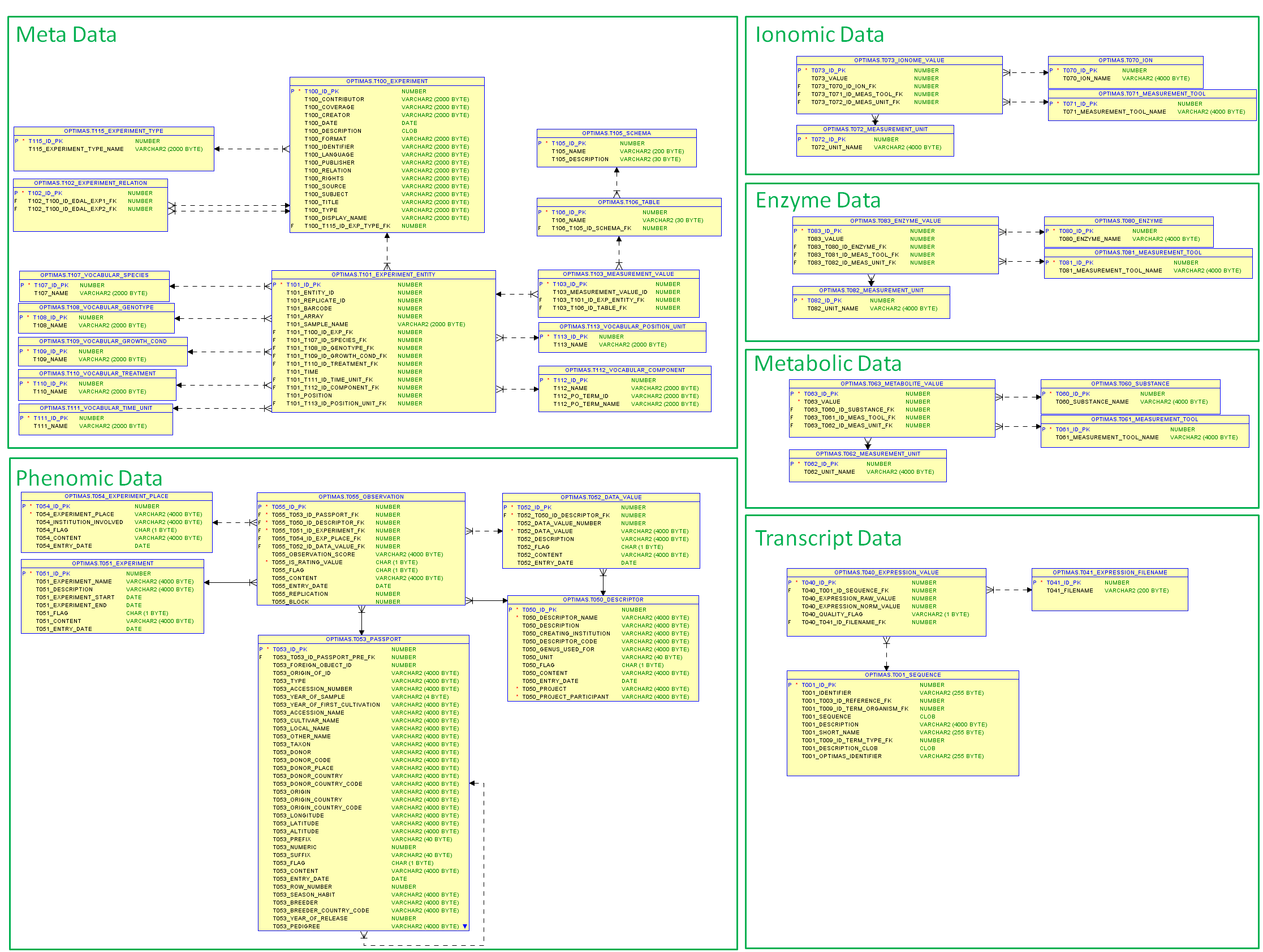

Supplement: Additional file 1 — OPTIMAS Database Schema. The OPTIMAS Database Schema can be divided into two main parts, the metadata on the one side and the data domain schemas on the other side. The metadata and each data domain schema are linked through table optimas.t103_measurement_ value. The primary key of the data domain entry is stored in t103_ measurement_value_id while the information about schema and table is stored in the related tables. [file 1471-2229-12-245-S1.PNG]
